# Variants in *Lrrk2* and *Snca* deficiency do not alter the course of primary encephalitis due to neurotropic reovirus T3D in newborn mice

**DOI:** 10.1371/journal.pone.0325248

**Published:** 2025-06-05

**Authors:** Michaela O. Lunn, Christopher Rousso, Julianna J. Tomlinson, Earl G. Brown, Michael G. Schlossmacher

**Affiliations:** 1 Program in Neuroscience, Ottawa Hospital Research Institute, Ottawa, Ontario, Canada; 2 Department of Cellular and Molecular Medicine, University of Ottawa, Ottawa, Ontario, Canada; 3 Department of Biochemistry, Microbiology, and Immunology, University of Ottawa, Ottawa, Ontario, Canada; 4 University of Ottawa Brain & Mind Research Institute, Ottawa, Ontario, Canada; 5 Division of Neurology, Department of Medicine, The Ottawa Hospital, Ottawa, Ontario, Canada; University College London, UNITED KINGDOM OF GREAT BRITAIN AND NORTHERN IRELAND

## Abstract

Variants at the leucine-rich repeat kinase-2 (*LRRK2*) and α-synuclein (*SNCA*) loci are associated with Parkinson’s disease (PD) risk. Viral infections have also been linked to increased risk of developing PD. In exploring a role for each of the encoded proteins in host response against brain-directed viral infections, we previously demonstrated that two *Lrrk2* knock-in variants as well as *Snca* expression altered survival rates from viral encephalitis following intranasal inoculation of newborn mice with a double-stranded RNA virus: respiratory-enteric-orphan virus, serotype-3 strain Dearing (reovirus T3D). Here, we examined whether outcomes of direct inoculation of the brain by reovirus T3D, which invariably causes lethal encephalitis within 15 days, would also be modified by variants in *Lrrk2* and *Snca*. When we inoculated newborn mice intracerebrally with reovirus T3D, we found that compared to wild-type littermates Lrrk2 p.G2019S kinase-hyperactive and p.D1994S kinase-inactive mutant mice did not show any significant difference in time-to-death or in viral titres in the brain, and revealed no sex difference. In parallel studies, the reduction or absence of endogenous α-synuclein did not alter the course of disease in reovirus T3D-infected mice. Together, these findings suggest that while variants in the PD-linked *Lrrk2* and *Snca* genes influenced disease outcomes of intranasally acquired reovirus T3D encephalitis, they did not affect survival outcomes in the intracerebrally acquired reovirus T3D encephalitis model.

## Introduction

Parkinson’s disease (PD) is one of the most prevalent neurodegenerative disorders, characterized in part by the progressive loss of dopaminergic neurons in the Substantia nigra pars compacta, leading to its typical movement disorder. While the exact etiology of PD remains elusive, its pathogenesis is thought to require a multifaceted interplay between genetic and environmental factors. Initiation of PD pathology may begin outside of the brain, such as in the nose and/or gut, two sites that are in direct contact with the external environment [1,2]. Consistent with a theory that PD could result in part from environmental — possibly microbial — triggers, epidemiological data suggest that the incidence rate of prior infections with one of several viruses, including hepatitis C virus, herpes simplex virus, and influenza A virus, is significantly correlated with elevated PD risk [3]. There is increasing interest in elucidating the role of environmental triggers and the contribution of both peripheral and nervous system-specific host responses in the initiation and progression of PD pathology.

*LRRK2* and *SNCA* are two loci linked to altered risk regarding the development of PD. We and others have shown in mice that both genes are involved in host responses to viral and bacterial infections [4–12]. Furthermore, we found that the PD-linked p.G2019S mutation in *Lrrk2* conferred a hyper-inflammatory phenotype in infected mice, leading to better control of both *Salmonella typhimurium* growth and reovirus serotype-3 strain Dearing (T3D) titres following intravenous and intranasal inoculation, respectively. However, when the infection reached the brain in the case of reovirus T3D, despite lower titres, Lrrk2 p.G2019S knock-in mice had worse health outcomes indicated by increased mortality, which was associated with a significant female sex bias. Contrarily, Lrrk2 kinase-dead p.D1994S neonates were relatively protected from intranasal infection by reovirus T3D, with increased survival rates when compared to their wild-type littermates, suggesting a deleterious gain-of-function effect for the Lrrk2 p.G2019S mutation.

Both *Lrrk2* and *Snca* are expressed in the brain and the periphery. Host immunity involves mechanisms both outside and inside the nervous system, with communication between the two. Our previous experiments investigated the role(s) of Lrrk2 and α-synuclein in a model that had engaged both peripheral and CNS-based immune responses [4,10]. The relative contribution of these proteins in host defences against reovirus T3D infection-induced encephalitis in peripheral organs versus the brain remained unknown. As such, we sought to investigate the contribution of these genes in a CNS-targeted, viral infection model, minimizing peripheral immune system involvement; to this end, we measured *Lrrk2*- and *Snca*-dependent outcomes following a direct-brain inoculation of murine pups with reovirus T3D. Intriguingly, neither *Lrrk2* variants nor *Snca* expression altered acute disease outcomes, including time-to-death intervals and viral titres following direct-brain inoculation.

## Methods

### Mouse colonies

Our animal experiments were performed in accordance with the guidelines of the Canadian Council on Animal Care; the study was approved by the University of Ottawa Animal Care Committee. Experiments involving viral infections were done in a containment level 2 biohazard animal facility. Lrrk2 p.G2019S mice and Lrrk2 p.D1994S mice were generated, as described in [13]; α-Synuclein knockout (*Snca*^*-/-*^) mice were first described in [14] and obtained from the Dr. Matthew Farrer lab. All mice are bred on a C57Bl/6J background. Both female and male mice were used in this study, as indicated within each figure.

### Intracerebral Reovirus T3D infection

From heterozygous breeding pairs, post-natal day 1 (p1) mouse pups were anesthetized under 3% isoflurane in oxygen. Using a 30-gauge 50 μL fixed needle Hamilton syringe, 10 μL of live, purified Respiratory Enteric Orphan Serotype 3 Dearing Virus (reovirus T3D) in PBS at a dose of 5x10^2^ PFU, or 10 μL of PBS alone, was injected into the left hemisphere of the pup brain (frontal lobe) [15] and returned to the home cage. Reovirus T3D (Fields strain) was provided by Dr. Earl Brown at the University of Ottawa [16].

### Intracerebral Reovirus T3D infection survival assay

Infected animals were assessed for survival, measured as time-to-death, using humane endpoints. The pre-determined humane endpoint due to encephalitis was characterized by immobility, neurological signs such as darting and myoclonus-type leg movements, and/or sustained lethargy. Mice were monitored twice daily (early morning and evening) up to 14 days post inoculation (dpi). A sample size of n = 226 was used for time-to-death analyses and mice were euthanized via rapid decapitation at the humane endpoint; a subset of mice (~15%), although not moribund during the evening health check, succumbed to the infection overnight and were found dead at the early morning health check.

### Viral quantification in tissue

A separate cohort of infected mice were euthanized at 8dpi via rapid decapitation for viral titre analyses of their brain and liver. Samples in PBS at a 3:1 ratio (μl PBS:mg tissue) for brains, or a 5:1 ratio for livers, were homogenized with metal beads using a Roche MagNA Lyser (Roche, Indianapolis, IN, USA) at maximum speed for 10 seconds. Samples underwent a freeze-thaw cycle in liquid nitrogen and were then centrifuged to collect the viral content in the supernatant for standard reovirus plaque assay analysis as described in [4]. Briefly, tissue homogenates were used for serial dilutions and overlaid onto L929 cells (originally obtained from the Dr. B. N. Fields lab) in 6-well plates at 90–100% confluency. Infected cells were overlaid with 2% purified agar and 199 media (2x) in a 1:1 ratio. Plaques were identified after 7 days of incubation using 0.015% neutral red stain and manually counted. PFU was reported as number of plaques per gram of tissue.

### Statistical analysis

All statistical analyses were performed using GraphPad Prism version 8 (GraphPad Software, San Diego, CA, USA, www.graphpad.com). Differences between groups in survival assays were assessed using the log-rank (Mantel-Cox) test. Differences between groups were assessed using one-way ANOVA with Tukey’s post-hoc tests. Data are demonstrated as mean ± SEM where applicable and as described in the figure legends. Data are displayed with *P* values and represented as * *P* < 0.05 for statistical significance.

## Results

### Parkinson’s-linked Lrrk2 p.G2019S variant does not alter acute outcomes in primary reovirus T3D-induced encephalitis model

**Fig 1:**
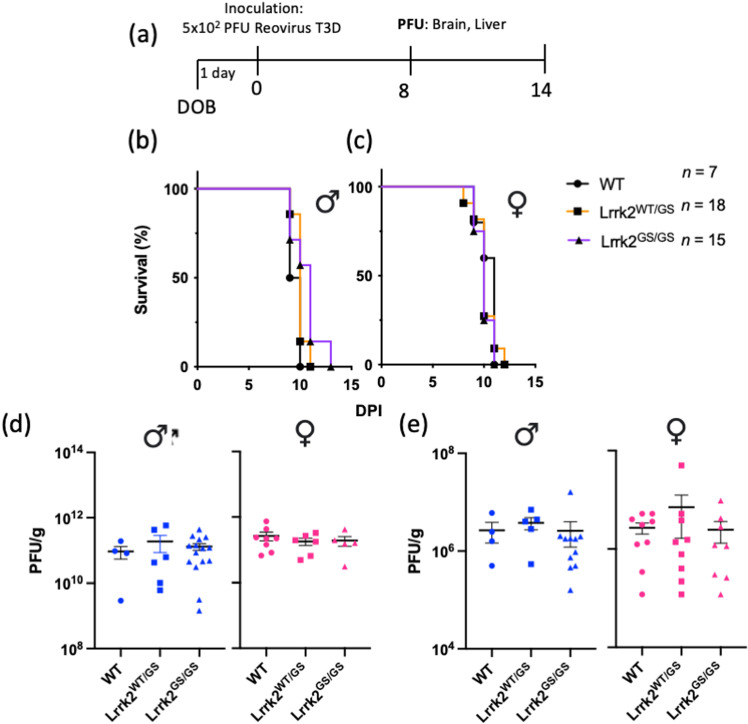
Expression of the Parkinson’s Disease-Linked Lrrk2 p.G2019S Variant Does Not Alter Outcomes of Acute Reovirus T3D-Induced Encephalitis Following Intracerebral Inoculation. Heterozygous Lrrk2 p.G2019S-carrying breeders were paired. Newborn mice were inoculated with a dose of 5x10^2^ PFU of reovirus T3D via direct brain injection into the left hemisphere and monitored for survival or sacrificed for tissue collection, as summarized in (**a**). Inoculated pups were monitored over 14 days for a pre-determined moribund state. Time-to-death is graphically displayed as percent survival against days post-inoculation (dpi), separated by sex (**b, c**). The n value indicates the sample size of sexes combined. The sample sizes of male mice were WT n = 2, Lrrk2^WT/DS^ n = 7 and Lrrk2^DS/DS^ n = 7. The sample sizes of female mice were WT n = 5, Lrrk2^WT/GS^ n = 11 and Lrrk2^GS/GS^ n = 8. In a second cohort, inoculated pups were sacrificed at 8dpi; brain and liver were collected and homogenized. Viral titres of the brain (**d**) and the liver (**e**) were measured via plaque assay. Data are represented in plaque-forming units (PFU)/g, where each symbol represents one animal. Blue data points indicate male mice (*n* ≥ 4 per group) and pink data points indicate female mice (*n* ≥ 5 per group). Survival curves were statistically analyzed using Mantel-Cox (Log-Rank) tests; viral titres were analyzed using one-way ANOVA and Tukey post-hoc tests. No significant group differences were observed.

The Lrrk2 p.G2019S variant was examined for its impact on acute outcomes in reovirus T3D-induced encephalitis following intracerebral inoculation. Of note, unlike the intranasal inoculation route, which mouse pups can survive, intracerebral inoculation with reovirus T3D is invariably lethal [17]. Lrrk2 p.G2019S pups at postnatal day 1 were injected intracerebrally with 5x10^2^ PFU reovirus T3D and either monitored for time-to-death or used for tissue collection and viral titre analyses (**Fig 1a**). Mice carrying the p.G2019S allele (heterozygous or homozygous) had similar time-to-death as did wild-type mice (*n* ≥ 7 mice per group) (**Fig 1b**, **1c**). Previously, we observed a female sex bias with Lrrk2 in infectious disease states; therefore, we analyzed the data separately based on sex, but we observed no difference between male and female animals (**Fig 1b**, **1c**).

To assess viral infectivity and its replication in the brain, we quantified viral titres at 8 days post-infection (dpi), as the latest timepoint before mice begin to succumb to the infection. Viral titres in the brain were similar among all genotypes, regardless of sex (**Fig 1d**). We also investigated secondary dissemination of the pathogen from the brain by measuring viral titres in the liver at 8dpi and observed viral spread from the brain to the periphery, as expected. However, there were no differences in viral titres between wild-type and Lrrk2 p.G2019S-expressing mice (**Fig 1e**). Collectively, these findings indicated that the Lrrk2 p.G2019S hyper-kinase mutation did not alter the course of acute illness in pups following intracerebral inoculation with reovirus T3D.

### Lrrk2 kinase-inactive mice show equal survival rate as heterozygous and wild-type animals following direct brain inoculation with reovirus T3D

**Fig 2:**
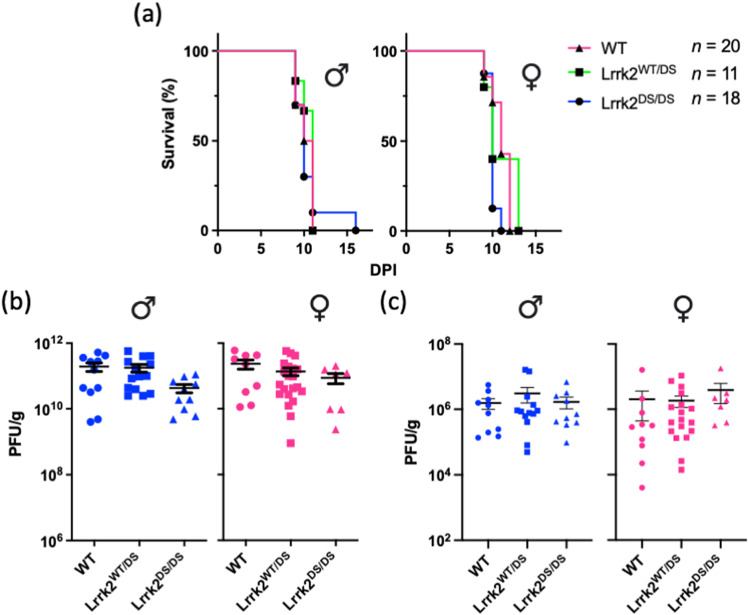
Expression of the Lrrk2 Kinase-Dead p.D1994S Mutant Does Not Alter Outcomes of Acute Reovirus T3D-Induced Encephalitis Following Intracerebral Inoculation. Heterozygous Lrrk2 p.D1994S-carrying breeders were paired. Newborn mice were inoculated with a dose of 5x10^2^ PFU of reovirus T3D via direct brain injection into the left hemisphere. Inoculated pups were monitored over 14 days for a pre-determined moribund state. Time-to-death is graphically displayed as percent survival against days post inoculation (DPI), separated based on sex (**a**). The *n* value indicates the sample size of sexes combined. The sample sizes of male mice were WT n = 10, Lrrk2^WT/DS^ n = 6 and Lrrk2^DS/DS^ n = 10. The sample sizes of female mice were WT n = 10, Lrrk2^WT/DS^ n = 5 and Lrrk2^DS/DS^ n = 8. In a second cohort, inoculated pups were sacrificed at 8dpi; brain and liver were collected and homogenized. Viral titres of the brain (**b**) and liver (**c**) were measured via plaque assay. Data are represented in plaque-forming units (PFU)/g, where each symbol represents one animal. Blue data points indicate male mice (*n* ≥ 9 per group) and pink data points indicate female mice (*n* ≥ 7 per group). Survival curves were statistically analyzed using Mantel-Cox (Log-Rank) tests and viral titre data were statistically analyzed using one-way ANOVA and Tukey post-hoc tests. No significant group differences were observed.

We next investigated whether Lrrk2’s kinase activity was required for the host’s defence against intracerebral inoculation with reovirus T3D; thus, we conducted the same experiments as above, but in kinase-dead Lrrk2 p.D1994S knock-in mice [13]. Unlike the survival benefit conferred by this mutation following intranasal inoculation [4], there was no difference in time-to-death between wild-type, heterozygous, and homozygous Lrrk2 p.D1994S mice (*n* ≥ 13 mice per group) following direct brain inoculation (**Fig 2a**). Consistent with this, Lrrk2 p.D1994S mice had similar viral titres in the brain at 8dpi compared to wild-type and heterozygous mice (**Fig 2b**). As expected, we observed viral dissemination to the liver at 8dpi in the Lrrk2 p.D1994S cohort, with relatively low but comparable viral titres across the three genotypes (**Fig 2c**). Collectively, these findings suggested that neither p.D1994S nor p.G2019S allelic variants in Lrrk2 influenced the course of encephalitis after direct injection of reovirus T3D into the brain.

### Endogenous α-synuclein does not protect against intracerebrally acquired reovirus T3D-induced encephalitis

**Fig 3:**
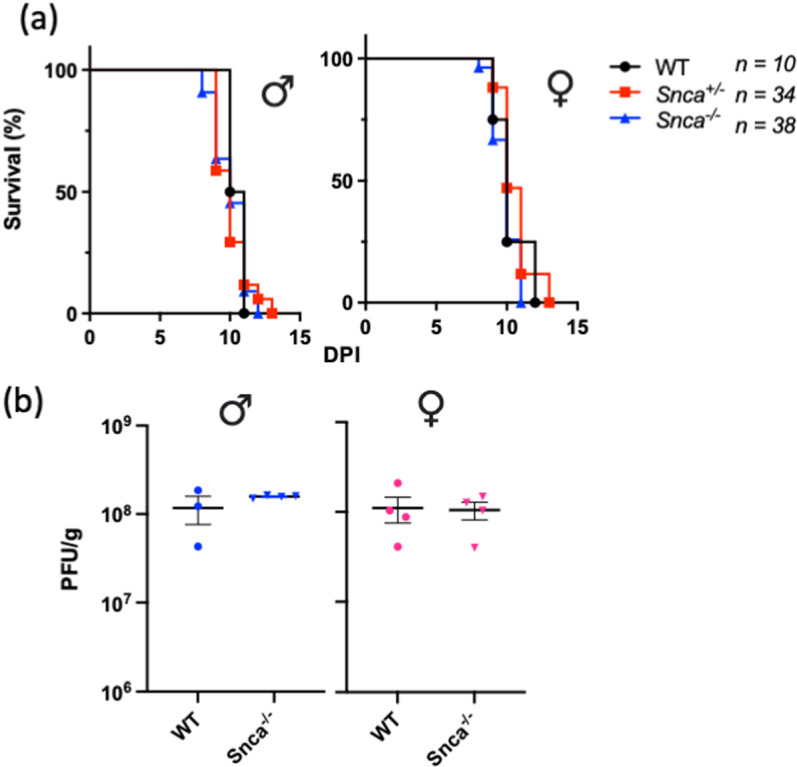
Expression of Murine *Snca* Gene Does Not Alter Outcomes of an Acute, Intracerebral Infection by Reovirus T3D-Induced Encephalitis. Newborn wild-type (WT), *Snca* knock-out (*Snca*^-/-^), and heterozygous (*Snca*^+/-^) pups were inoculated with a dose of 5x10^2^ PFU of reovirus T3D via direct brain injection into the left hemisphere. Inoculated pups were monitored over 14 days for a pre-determined moribund state. Time-to-death is graphically displayed as percent survival against days post inoculation (DPI), separated based on sex (**a**). The *n* value indicates the sample size of sexes combined. The sample sizes of male mice were WT n = 6, Snca^+/-^ n = 17 and Snca^-/-^ n = 11. The sample sizes of female mice were WT n = 4, Snca^+/-^ n = 17 and Snca^-/-^ n = 27. In a second cohort, inoculated pups were sacrificed at 8dpi and brains were collected for homogenization. Viral titres of the brain were measured via plaque assay (**b**). Data are represented in plaque-forming units (PFU)/g, where each symbol represents one animal. Blue data points indicate male mice (*n* ≥ 3 per group) and pink data points indicate female mice (*n* ≥ 4 per group). Survival curves were statistically analyzed using Mantel-Cox (Log-Rank) tests and viral titre data were statistically analyzed using one-way ANOVA and Tukey post-hoc tests. No significant group differences were observed.

Akin to Lrrk2, and building on our previous discovery that *Snca* expression was protective against intranasal inoculation of pups with reovirus T3D [10], we further investigated the antiviral properties of α-synuclein to protect against virus-induced encephalitis. We performed direct brain inoculation of reovirus T3D in *Snca*^-/-^, *Snca*^+/-^ and wild-type littermates. In contrast to intranasal, systemic administration of reovirus T3D, direct brain inoculation did not result in any genotypic differences in time-to-death, regardless of sex (*n* ≥ 10 mice per group) (**Fig 3a**). Similarly, *Snca* expression also did not affect viral replication in the brain at 8dpi, regardless of sex (**Fig 3b**).

### A lower dose of reovirus T3D and older age at inoculation does not reveal genotypic differences in time-to-death of resultant infection in mutant mice

A key difference between intranasal and intracerebral inoculation models for reovirus T3D in suckling pups is that while the lethality of the infection following intranasal inoculation is titre-dependent, the median lethal dose of reovirus T3D delivered via direct inoculation of the brain is 10^1^ PFU [17–19]. This represents a limitation of our study, in that the severity of the intracerebral inoculation method may be too fulminant to reveal time-to-death differences between genotypes in contrast to the intranasal reovirus T3D infection paradigm.

**Fig 4:**
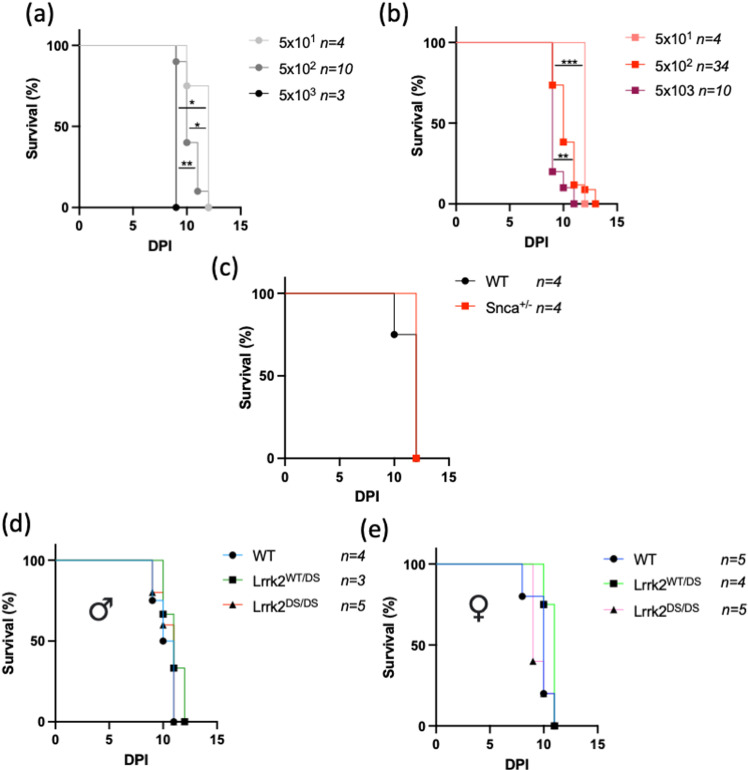
Neither a Lower Dose Nor Inoculating Mice At Post Natal Day 3 Uncovers Genotypic Differences in Time-To-Death of Intracerebral Inoculation of Reovirus T3D. Newborn wild-type (WT) (**a**) and heterozygous *Snca* null (Snca^+/-^) (**b**) mice were inoculated with either 5x10^1^ PFU, 5x10^2^ PFU, or 5x10^3^ PFU of reovirus T3D via intracerebral injection at p1. Inoculated pups were monitored over 14 days for a pre-determined moribund state. Time-to-death is graphically displayed as percent survival against days post inoculation (DPI). Survival curves of wild-type and heterozygous *Snca* null (Snca^+/-^) mice infected with a dose of 5x10^1^ PFU were compared, and no statistical differences were found (**c**). Next, wild-type, Lrrk2 p.D1994S heterozygous (Lrrk2^WT/DS^), and Lrrk2 p.D1994S homozygous (Lrrk2^DS/DS^) mice at post-natal day 3 were inoculated with a dose of 5x10^2^ PFU of reovirus T3D via intracerebral injection. Time-to-death is graphically displayed as percent survival against days post inoculation (DPI), for male (**d**) and female (**e**) mice. The *n* values are indicated within the figure. Survival curves were statistically analyzed using Mantel-Cox (Log-Rank) tests. No significant group differences were observed.

To address this limitation, we investigated the outcomes of three doses of reovirus T3D in wild-type (**Fig 4a**) and heterozygous *Snca*^+/-^ mice (**Fig 4b**): 5x10^1^ PFU, 5x10^2^ PFU, and 5x10^3^ PFU. In both wild-type and heterozygous *Snca*^+/-^ mice, each dose was uniformly lethal; however, the assay proved sensitive enough to detect significant changes in the time-to-death intervals. This outcome suggested to us that this model employs a challenge dosage within a responsive range that is sensitive to change and thus suitable for detecting/assessing genotypic effects of either *Lrrk2* or *Snca* mutants, similar to those that were observed in the nasally-inoculated, respiratory infection model, where a much higher dosage was employed (1.7x10^5^ PFU/mouse, to achieve a 50% lethality) [4,10]. Furthermore, when comparing the time-to-death outcomes of wild-type and heterozygous *Snca*^+/-^ mice at the lowest dose, i.e., 5x10^1^ PFU, we did not observe any difference between the two genotypes (**Fig 4c**), matching the data when using the standard dose of 5x10^2^ PFU (**Fig 3a**).

Given that when administered via the intranasal route, the viral infection reaches the brain only two days post-inoculation, we next sought to determine whether post-natal time of infection could affect the response of the survival assay. As such, we inoculated a cohort of mutant Lrrk2 p.D1994S mice at 3 days after birth intracerebrally with 5x10^2^ PFU of reovirus T3D, but time-to-death intervals were identical to those infected on postnatal day 1, with no differences observed between the genotypes or between sexes (**Fig 4d**, **4e**).

## Discussion

Host responses to microbial infections are pathogen- and tissue-dependent. We have previously shown that two PD-linked genes, *Lrrk2* and *Snca*, confer anti-microbial functions in mice. In the case of Lrrk2, we (and others) showed that this occurred in a pathogen-, tissue-, and sex-dependent manner. Specifically, while female Lrrk2 p.G2019S mice were better able to control viral titres and *Salmonella typhimurium* burden in peripheral organs, in the case of intranasal inoculation with reovirus T3D, which subsequently infected the brain, mutant mice had greater disease severity with decreased survival due to encephalitis. Both of these genes are expressed within the CNS – including in neurons and microglia – as well as in peripheral organs. Immune responses that occur in the periphery can be critical to brain health. In this current study, we sought to determine whether Lrrk2 and α-synuclein also contribute to the immediate antiviral host response as relates to survival outcomes from encephalitis following direct inoculation of the brain. Intranasal and intracerebral inoculations of murine pups cause lethal encephalitis as a result of infection of the brain following a similar time course of disease but via different routes [16].

Surprisingly, in this study neither allelic variants in *Lrrk2* nor *Snca* expression impacted survival or viral replication following intracerebral inoculation. Taken together, these data suggest that both genes likely contribute to peripheral host responses to reovirus T3D, and that it may be peripheral organ involvement that conferred partial neuroprotection from otherwise lethal encephalitis when the infection had started outside the nervous system. When the virus was directly injected into the brain, these genes had no impact on time-to-death, nor viral titres. Notably, both intranasal and intracerebral inoculation of reovirus T3D resulted in similar symptomatology and illness progression, with peak lethality occurring between 6–10dpi in both models.

Recent literature supports that Lrrk2 may influence pathogenic outcomes in the brain through peripheral involvement rather than functioning directly in the brain. It has been demonstrated not only that the peripheral administration of a bacterial antigen, lipopolysaccharide (LPS), could drive dopaminergic neuronal loss in transgenic Lrrk2-mutant mice (p.R1441G and p.G2019S), but also that it was dependent on the presence of peripheral lymphocytes; further, neutralization of peripheral IL-6 prevented the LPS-induced cell loss in transgenic, Lrrk2-mutant mice [20]. These findings by the Smeyne laboratory support the hypothesis that allelic variants in *Lrrk2* may exert modulatory effects on immune cells and cytokines in the periphery, which in turn confer secondary, deleterious effects on dopaminergic neuronal health.

Akin to Lrrk2, albeit less-studied in this context, α-synuclein has been reported to have anti-microbial properties both *in vivo* and *in vitro,* including to protect mice from viral and bacterial infections that have begun outside the central nervous system [10,11]. Furthermore, α-synuclein has been shown to be involved in generating inflammatory responses to pathogens in the periphery [8]. Increased *Snca* expression and post-translational modification have been reported downstream of microbial antigen exposures [21–23], suggesting that the gene and encoded protein may respond to environmental triggers as part of a physiological role in host response; these findings could also link environmental exposures and ensuing host responses to disease-relevant changes in α-synuclein metabolism.

The primary goal of our study was to determine whether Lrrk2 and α-synuclein function within the brain to protect mice from acute reovirus T3D-induced encephalitis. Unexpectedly, they did not. When taken together with our previous studies in which the same virus was administered intranasally and led to genotype-dependent survival benefits [4,10], these findings suggest to us that both of these genes could contribute to neuroprotection, at least in part, by contributing to peripheral immune responses. A limitation of directly comparing these two paradigms, however, is that while intranasal inoculation can be titrated to non-lethality, intracerebral inoculation is invariably lethal at any dose [17]. Nonetheless, we confirmed that intracerebral administration of 5x10^2^ PFU was within a dose range that was sensitive to titre-dependent changes in time-to-death and that a dose of 5x10^1^ PFU would likely not uncover further differences between groups. Further, employing a time of inoculation which better matched the age at which the virus enters the brain in the intranasal model (2dpi) did not impact the outcomes of the primary study. It is notable that following intranasal inoculation, the viral titres of reovirus T3D in the brain at 2dpi are in the range of 10^5^ PFU/g [16], which is much higher than the intracerebral lethal dosage used in this study, but the former dose results in only 50% lethality in wild type mice due to factors that mediate host resistance occurring outside of the brain.

Future work will seek to identify the host factors and/or responses that are activated by intranasal infection to modify the severity of subsequent encephalitis in a *Lrrk2*- and *Snca*-dependent manner, respectively. These experiments will directly assess the effects of prior respiratory infection on delayed intracerebral infections; alternatively, factors may be identified and passively transferred or modulated in the intracerebral model. Finally, in order to directly compare the results of the intranasal versus the intracerebral inoculation models, a full investigation and profiling of the viral pathogenesis and host responses in both models are required.

While this viral model has allowed us to investigate a role for these proteins in an acute, innate immunity driven infection course, assessments of Lrrk2- and α-synuclein-based anti-viral properties in adult mice are ongoing and will allow us to investigate their role in a mature immune system. There, we are able to study their roles in the context of multiple critical factors, including age as a determinant in disease progression, development beyond puberty and the influence of sex hormones, as well as the maturation of an adaptive immune response. In addition to this, testing of other types of viruses, such as DNA- *vs.* RNA-based pathogens (including those with double-stranded *vs.* single-stranded genomes), in the context of *Snca* and *Lrrk2* mutants is warranted.

Taken together with our previous publications using the same virus (strain, dose) and same animal models [4,10], this study suggests that while the PD-associated genes *Lrrk2* and *Snca* contribute to host defense against reovirus T3D-induced encephalitis when acquired systemically, they do not influence disease outcomes (*i.e.,* time-to-death and viral titres) when the virus is administered directly to the brain. These findings contribute to the increasing body of evidence indicating that both Lrrk2 and α-synuclein may have physiological roles outside the brain, including those that may contribute to overall nervous system health through systemic immunity. Further modeling of the numerous and complex interactions between environmental exposures and genetic risk factors in peripheral organs is warranted when studying the pathogenesis of PD.

## References

[1] Kalia LV, Lang AE. Parkinson’s disease. Lancet. 2015;386(9996):896–912. doi: 10.1016/S0140-6736(14)61393-3 25904081

[2] Braak H, Del Tredici K, Rüb U, de Vos RAI, Jansen Steur ENH, Braak E. Staging of brain pathology related to sporadic Parkinson’s disease. Neurobiol Aging. 2003;24(2):197–211. doi: 10.1016/s0197-4580(02)00065-9 12498954

[3] Leta V, Urso D, Batzu L, Lau YH, Mathew D, Boura I, et al. Viruses, parkinsonism and Parkinson’s disease: the past, present and future. J Neural Transm (Vienna). 2022;129(9):1119–32. doi: 10.1007/s00702-022-02536-y 36036863 PMC9422946

[4] Shutinoski B, Hakimi M, Harmsen IE, Lunn M, Rocha J, Lengacher N, et al. Lrrk2 alleles modulate inflammation during microbial infection of mice in a sex-dependent manner. Sci Transl Med. 2019;11(511):eaas9292. doi: 10.1126/scitranslmed.aas9292 31554740

[5] Härtlova A, Herbst S, Peltier J, Rodgers A, Bilkei-Gorzo O, Fearns A, et al. LRRK2 is a negative regulator of Mycobacterium tuberculosis phagosome maturation in macrophages. EMBO J. 2018;37(12):e98694. doi: 10.15252/embj.201798694 29789389 PMC6003659

[6] Liu W, Liu X, Li Y, Zhao J, Liu Z, Hu Z, et al. LRRK2 promotes the activation of NLRC4 inflammasome during Salmonella Typhimurium infection. J Exp Med. 2017;214(10):3051–66. doi: 10.1084/jem.20170014 28821568 PMC5626397

[7] Zhang Q, Pan Y, Yan R, Zeng B, Wang H, Zhang X, et al. Commensal bacteria direct selective cargo sorting to promote symbiosis. Nat Immunol. 2015;16(9):918–26. doi: 10.1038/ni.3233 26237551

[8] Alam MM, Yang D, Li X-Q, Liu J, Back TC, Trivett A, et al. Alpha synuclein, the culprit in Parkinson disease, is required for normal immune function. Cell Rep. 2022;38(2):110090. doi: 10.1016/j.celrep.2021.110090 35021075 PMC10258816

[9] Monogue B, Chen Y, Sparks H, Behbehani R, Chai A, Rajic AJ, et al. Alpha-synuclein supports type 1 interferon signalling in neurons and brain tissue. Brain. 2022;145(10):3622–36. doi: 10.1093/brain/awac192 35858675 PMC10233298

[10] Tomlinson JJ, Shutinoski B, Dong L, Meng F, Elleithy D, Lengacher NA, et al. Holocranohistochemistry enables the visualization of α-synuclein expression in the murine olfactory system and discovery of its systemic anti-microbial effects. Journal of Neural Transmission. 2017 Jun 5;124(6):721–38. 28477284 10.1007/s00702-017-1726-7 PMC5446848

[11] Beatman EL, Massey A, Shives KD, Burrack KS, Chamanian M, Morrison TE. Alpha-synuclein expression restricts RNA viral infections in the brain. J Virol. 2016;90(6):2767–82. 10.1128/JVI.02949-15 PMC4810656 26719256

[12] Chen SG, Stribinskis V, Rane MJ, Demuth DR, Gozal E, Roberts AM, et al. Exposure to the Functional Bacterial Amyloid Protein Curli Enhances Alpha-Synuclein Aggregation in Aged Fischer 344 Rats and Caenorhabditis elegans. Sci Rep. 2016;6:34477. doi: 10.1038/srep34477 27708338 PMC5052651

[13] Herzig MC, Kolly C, Persohn E, Theil D, Schweizer T, Hafner T, et al. LRRK2 protein levels are determined by kinase function and are crucial for kidney and lung homeostasis in mice. Hum Mol Genet. 2011;20(21):4209–23. doi: 10.1093/hmg/ddr348 21828077 PMC3188995

[14] Cabin DE, Shimazu K, Murphy D, Cole NB, Gottschalk W, McIlwain KL. Synaptic vesicle depletion correlates with attenuated synaptic responses to prolonged repetitive stimulation in mice lacking α-synuclein. J Neurosci. 2002;22(20):8797–807. 12388586 10.1523/JNEUROSCI.22-20-08797.2002 PMC6757677

[15] Passini MA, Wolfe JH. Widespread gene delivery and structure-specific patterns of expression in the brain after intraventricular injections of neonatal mice with an adeno-associated virus vector. J Virol. 2001;75(24):12382–92. doi: 10.1128/JVI.75.24.12382-12392.2001 11711628 PMC116134

[16] Gauvin L, Bennett S, Liu H, Hakimi M, Schlossmacher M, Majithia J, et al. Respiratory infection of mice with mammalian reoviruses causes systemic infection with age and strain dependent pneumonia and encephalitis. Virol J. 2013;10:67. doi: 10.1186/1743-422X-10-67 23453057 PMC3605257

[17] Tyler KL. Pathogenesis of reovirus infections of the central nervous system. Curr Top Microbiol Immunol. 1998;233(Pt 2):93–124. doi: 10.1007/978-3-642-72095-6_6 9599934

[18] Virgin HW 4th, Bassel-Duby R, Fields BN, Tyler KL. Antibody protects against lethal infection with the neurally spreading reovirus type 3 (Dearing). J Virol. 1988;62(12):4594–604. doi: 10.1128/JVI.62.12.4594-4604.1988 2460637 PMC253571

[19] Spriggs DR, Fields BN. Attenuated reovirus type 3 strains generated by selection of haemagglutinin antigenic variants. Nature. 1982;297(5861):68–70. doi: 10.1038/297068a0 6175910

[20] Kozina E, Byrne M, Smeyne RJ. Mutant LRRK2 in lymphocytes regulates neurodegeneration via IL-6 in an inflammatory model of Parkinson’s disease. NPJ Parkinsons Dis. 2022;8(1):24. doi: 10.1038/s41531-022-00289-9 35292674 PMC8924242

[21] Peelaerts W, Mercado G, George S, Villumsen M, Kasen A, Aguileta M, et al. Urinary tract infections trigger synucleinopathy via the innate immune response. Acta Neuropathol. 2023;145(5):541–59. doi: 10.1007/s00401-023-02562-4 36991261 PMC10119259

[22] Bantle CM, Phillips AT, Smeyne RJ, Rocha SM, Olson KE, Tjalkens RB. Infection with mosquito-borne alphavirus induces selective loss of dopaminergic neurons, neuroinflammation and widespread protein aggregation. NPJ Parkinsons Dis. 2019;5:20. doi: 10.1038/s41531-019-0090-8 31531390 PMC6744428

[23] Jang H, Boltz D, Sturm-Ramirez K, Shepherd KR, Jiao Y, Webster R, et al. Highly pathogenic H5N1 influenza virus can enter the central nervous system and induce neuroinflammation and neurodegeneration. Proc Natl Acad Sci U S A. 2009;106(33):14063–8. doi: 10.1073/pnas.0900096106 19667183 PMC2729020

